# Small-for-Gestational-Age Status and Adverse Clinical Outcomes in Preterm and Very Preterm Infants: A Propensity Score-Matched Cohort Study

**DOI:** 10.3390/jcm15124798

**Published:** 2026-06-20

**Authors:** Manapat Praditaukrit, Anucha Thatrimontrichai, Praew Chareesri, Pattima Pakhathirathien, Gunlawadee Maneenil, Supaporn Dissaneevate

**Affiliations:** Division of Neonatology, Department of Pediatrics, Faculty of Medicine, Prince of Songkla University, Songkhla 90110, Thailand; manapat.p@psu.ac.th (M.P.); praew.c@psu.ac.th (P.C.); ppattima@medicine.psu.ac.th (P.P.); gunlawadee.m@psu.ac.th (G.M.); dsupapor@medicine.psu.ac.th (S.D.)

**Keywords:** healthcare costs, infant mortality, multimorbidity, premature infant, small-for-gestational-age infant, very-low-birth-weight infant

## Abstract

**Background/Objectives:** Preterm (<37 weeks) and very preterm (<32 weeks) infants face considerably higher mortality and morbidity rates than full-term infants. We compared clinical outcomes between small-for-gestational-age (SGA) and appropriate-for-gestational-age (AGA) preterm infants. **Methods:** This retrospective cohort study used a prospectively collected database, obtained from 2014 to 2025. Propensity score matching (PSM), multivariate regression, and subgroup analyses of very preterm infants were performed to minimize confounding. **Results:** Among the 5890 neonatal admissions, 2331 preterm infants met the inclusion criteria. After PSM, 298 SGA and 298 AGA preterm infants were analyzed. Multivariate analysis showed that SGA preterm infants had significantly higher risks of the composite outcome of mortality or major morbidity (adjusted risk ratio [aRR], 1.89; 95% confidence interval [CI], 1.18–3.02), mortality (aRR, 3.53; 95% CI, 1.57–7.95), and mortality or moderate-to-severe bronchopulmonary dysplasia (aRR, 2.13; 95% CI, 1.30–3.48). In the subgroup analysis after PSM, 190 very preterm infants showed similar results, with SGA infants having increased risks of the composite outcome of mortality or major morbidity (aRR, 1.81; 95% CI, 1.02–3.23), mortality (aRR, 3.23; 95% CI, 1.09–9.62), mortality or moderate-to-severe bronchopulmonary dysplasia (aRR, 2.03; 95% CI, 1.10–3.72), and mortality or treated retinopathy of prematurity (aRR, 2.62; 95% CI, 1.03–6.65). **Conclusions:** SGA status is associated with a higher risk of mortality and major morbidity in preterm and very preterm infants. In resource-limited settings, the focused management of SGA infants is critical to improving short- and long-term outcomes.

## 1. Introduction

Preterm infants have higher rates of mortality and short- and long-term morbidities, including adverse cognitive and educational outcomes, than full-term infants [1]. Small-for-gestational-age (SGA), defined as a birth weight (BW) below the 10th percentile for gestational age (GA) and sex [2], is associated with significantly higher risks of metabolic and hematological complications, as well as long-term growth and neurodevelopmental deficits, than appropriate-for-gestational-age (AGA), defined as a BW between the 10th and 90th percentiles for GA and sex. On the first day of life, SGA infants have higher risks of hypoglycemia [3,4], hypothermia due to low glycogen and fat stores, and polycythemia due to chronic intrauterine hypoxia [4]. During hospitalization, SGA preterm infants often have higher risks of retinopathy of prematurity (ROP) [5,6,7,8], bronchopulmonary dysplasia (BPD) [5,6,8], patent ductus arteriosus [7], necrotizing enterocolitis (NEC) [3,4,5], intraventricular hemorrhage [7], and late-onset neonatal sepsis [4] than AGA infants.

At discharge, SGA status is associated with significantly higher neonatal mortality than that in AGA preterm infants [5,6]. SGA status is also strongly associated with longer durations of ventilation, exceeding 3 [5] or 18 days [8], and prolonged hospital stays [4], exceeding 10 [5], 13 [7], or 20 days [8], compared with AGA infants. At 2 years of age and beyond, SGA preterm infants are significantly more likely to remain underweight, remain shorter, and have smaller head circumferences than AGA children. Moreover, SGA preterm infants are at higher risk of neurodevelopmental impairment [6,9,10].

Previous studies of SGA preterm infants have primarily been conducted in high-income economies, including the USA [6,9], Canada [5,8], and Taiwan [11]. In contrast, published research from resource-limited settings remains scarce, with available data limited to specific cohorts in Brazil (GA < 37 weeks) [7], Ethiopia (GA 28–36 weeks) [4], and India (GA < 32 weeks) [3]. We aimed to evaluate clinical outcomes and neonatal morbidities among SGA preterm (GA < 37 weeks) and very preterm (GA < 32 weeks) infants admitted to our neonatal intensive care unit (NICU), using propensity score matching (PSM) to address these regional data gaps.

## 2. Methods

### 2.1. Study Design and Patient Population

This retrospective cohort study used prospectively collected data from the Songklanagarind Neonatal Database. The database captures clinical information from the level IV NICU [12] at Songklanagarind Hospital, which manages approximately 2500–3500 live births and 400–550 neonatal admissions, including both inborn and outborn cases, annually. To ensure data integrity, all clinical parameters in the database are predefined and extracted from primary medical records by trained statisticians.

The study population comprised preterm infants with a GA of less than 37 weeks who were born between 1 January 2014 and 31 December 2025, survived initial resuscitation, and were admitted to the NICU. Exclusion criteria included outborn infants, those classified as large-for-gestational-age (LGA), and infants with major congenital anomalies as defined by the Vermont Oxford Network [13].

The study protocol was approved by the Human Research Ethics Committee of the Faculty of Medicine, Prince of Songkla University (Approval No. 69-231-1-1). The requirement for informed consent was waived because of the retrospective study design and the use of de-identified data. NotebookLM (powered by Gemini 3) was used to assist with the conceptualization and structural development of the graphical abstract.

### 2.2. Exposure, Comorbidities, and Outcomes

The exposure of interest was birth SGA compared with AGA. SGA, AGA, and LGA were defined as BW below the 10th percentile, between the 10th and 90th percentiles, and above the 90th percentile, respectively, according to GA (week and day) and sex, based on the Fenton growth curves (2013) [2]. All maternal and neonatal data were extracted from the database. GA was determined by early ultrasonography or the last regular menstrual period.

Chorioamnionitis was defined by maternal fever greater than 38 °C and at least two of the following criteria: maternal leukocytosis, maternal tachycardia, fetal tachycardia, uterine tenderness, and/or foul-smelling amniotic fluid [14]. Persistent pulmonary hypertension in the newborn and patent ductus arteriosus were diagnosed by echocardiography and confirmed by a pediatric cardiologist, neonatologist, or trained neonatal fellow. Ventilator-associated pneumonia and central line-associated bloodstream infection were defined according to the National Healthcare Safety Network guidelines for infants aged less than 1 year, based on the version current at the time of diagnosis in the database [15,16]. Culture-proven sepsis was defined as clinical sepsis, indicated by clinical signs of infection accompanied by concurrent antibiotic treatment for at least 3 days, together with bacterial growth in at least one blood and/or cerebrospinal fluid culture for Gram-negative or Gram-positive bacteria, except for commensal organisms, which required at least two positive samples. NEC (stage II–III) was diagnosed according to modified Bell criteria, supported by clinical and radiographic findings [17].

The primary outcome was a composite outcome of mortality or major morbidity at discharge. Mortality was defined as in-hospital death before discharge. Major morbidity was defined as the presence of one or more of the evaluated morbidities: severe neurological injury, moderate-to-severe BPD, or treated ROP. Severe neurological injury was defined as intraventricular hemorrhage grade 3 or 4 and/or periventricular leukomalacia. Treated ROP was defined as treatment with either laser photocoagulation or intravitreal anti-vascular endothelial growth factor injection. Cranial ultrasonography and ophthalmologic examinations were performed only in infants with relevant indications. Moderate-to-severe BPD was defined as the need for supplemental oxygen for at least 28 days until 36 weeks of postmenstrual age or 56 days of life for infants born before or after 32 weeks of GA, respectively, or until discharge, whichever occurred first [18]. Length of hospital stay was defined as the duration of admission until discharge. Daily hospital cost was calculated as the total hospital cost divided by the length of hospital stay (1 U.S. dollar [USD] = 32 baht).

### 2.3. Sample Size Calculation

The definition of the composite outcome in the present study was consistent with that used in a US-based study conducted between 2010 and 2016 [6]. In contrast, other regional studies used broader definitions. For example, the composite outcome included NEC in a Canadian study [5] and both NEC and culture-proven sepsis in an Indian study [3].

Based on a previous US study [6], the composite outcome of mortality or major morbidity among SGA and AGA very preterm infants was reported to be 55.0% and 33.4%, respectively. For this cohort study, the sample size was calculated using a two-sided significance level of <0.05 and a statistical power of 80%. A minimum of 82 infants per group was required to detect a significant difference in the composite outcome between the SGA and AGA cohorts.

Based on our previous study conducted between 2014 and 2021 [19], the study center identified 80 SGA very preterm (GA <32 weeks) infants over an 8-year period, corresponding to approximately 10 infants per year. To achieve the target sample size of 82 infants per group, a study duration of at least 9 years was initially estimated. However, given the declining birth rate and overall reduction in preterm births, together with advances in neonatal care that may have reduced the incidence of composite outcomes in recent years, a longer study period was considered necessary. Furthermore, data on composite outcomes in SGA and AGA preterm (GA <37 weeks) infants remain limited. Accordingly, the study period was extended to 12 years (2014–2025) to ensure a sufficiently powered cohort for robust analysis.

### 2.4. Statistical Analysis

Statistical analyses were performed using STATA software (version SE 17; StataCorp LLC, College Station, TX, USA). Maternal characteristics, neonatal risk factors, and clinical outcomes were compared between the SGA and AGA cohorts for both preterm and very preterm infants. Categorical variables are presented as frequencies and percentages and were compared using the chi-square or Fisher exact test, as appropriate. Normally distributed data are presented as mean ± standard deviation and were compared using the unpaired *t*-test. Non-normally distributed data are presented as median (interquartile range) and were analyzed using the Mann–Whitney U test.

To evaluate chronological variations across the study period, the temporal trend of the primary composite outcome from 2014 to 2025 was analyzed using a generalized linear model (GLM) with a binomial family and identity link function, treating the year of birth as a continuous independent predictor.

### 2.5. Propensity Score Matching

To reduce potential confounding and selection bias inherent in this observational design, PSM was utilized. Propensity scores—representing the conditional probability of being born SGA—were estimated using a binary logistic regression model. Following established causal inference principles, baseline maternal and neonatal covariates were selected if they were strong prognostic factors for the primary outcomes based on clinical plausibility or exhibited a standardized mean difference (SMD) ≥ 0.1 in the univariate analysis of the overall cohort. The list of covariates incorporated into the PSM models comprised GA, male sex, antenatal steroid exposure, and year of birth, together with variables showing a SMD > 0.1 in the univariate analysis (multifetal gestation, Cesarean delivery, and pregnancy-induced hypertension for both the overall preterm and very preterm analyses, and chorioamnionitis and 5 min Apgar score for very preterm analyses).

Matching was executed using the iterative stratification and stochastic optimization routine via the mapsm command in Stata. Estimated propensity scores were categorized into 10 equal strata (e.g., 0–0.1, 0.1–0.2, up to 0.9–1.0), and participants were matched in a 1:1 ratio within their respective strata. To achieve optimal balance, the matching procedure was repeated for 200 iterations. The final post-matched cohorts were selected based on the iteration yielding the lowest overall SMDs, indicating the highest degree of covariate balance. A random-number seed value of 1522 was specified for both groups to ensure exact reproducibility of the optimal matched sample.

**Figure 1:**
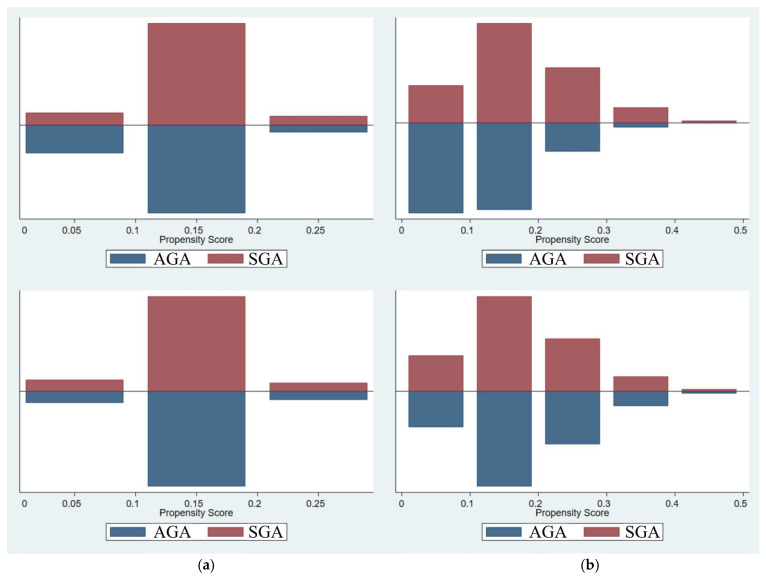
Propensity score overlap plots for the small-for-gestational-age (SGA) and appropriate-for-gestational-age (AGA) cohorts. The mirrored histograms illustrate the distribution of estimated propensity scores for the SGA (top/red bars) and AGA (bottom/blue bars) groups. Panels show the baseline (upper panels) and post-matching (lower panels) distributions for (**a**) the overall preterm infant cohort and (**b**) the very preterm infant subgroup.

Post-matching covariate balance was assessed using SMDs, with a conservative threshold of <0.1 prespecified to indicate negligible baseline imbalance between the cohorts. The distributions of propensity scores before and after matching were visually verified using mirrored histograms (Figure 1a,b).

To identify variables independently associated with outcomes in the matched cohorts, multivariate regression models were constructed. Adjusted risk ratios were estimated using GLM with a log link and Poisson distribution family, utilizing robust error variances to handle binary outcomes. Adjusted risk differences were calculated using a linear identity link within the GLM framework. The adjusted risk ratios and adjusted risk differences in the final models represent the controlled direct effects of SGA status, rather than the total causal effect.

To mitigate the potential for over-regression and model overfitting given the rare nature of certain composite events, variable selection for the final multivariable models was highly constrained. Rather than adjusting for all available clinical parameters, covariates were limited strictly to the primary exposure (SGA status) and pre-exposure baseline variables that retained a univariate *p*-value <0.2, adhering to a conservative events-per-variable threshold to maintain statistical stability and ensure valid confidence intervals.

## 3. Results

During 2014–2025, a total of 5890 infants were admitted to the NICU. After the application of the exclusion criteria, term infants (*n* = 2798), outborn infants (*n* = 514), LGA infants (*n* = 103), and infants with major congenital anomalies (*n* = 144) were excluded. The final cohort therefore comprised 2331 preterm infants, of whom 298 (12.8%) were SGA.

Over the 12-year study period, the annual incidence of the primary composite outcome (mortality or major morbidity) demonstrated a stable baseline trajectory without a statistically significant linear trend. Specifically, the annual rates from 2014 to 2025 were 13.0%, 9.6%, 9.3%, 14.8%, 14.0%, 10.4%, 10.5%, 10.0%, 10.9%, 10.0%, 8.7%, and 10.0%, respectively (regression coefficient = −0.02, *p* = 0.26).

### 3.1. Preterm Infants

Maternal and neonatal covariates associated with the composite outcome, including GA, male sex, antenatal steroid exposure, and year of birth, together with variables showing SMD >0.1 in the univariate analysis (multifetal gestation, Cesarean delivery, and pregnancy-induced hypertension), were included in the PSM model. After matching, covariate balance was achieved between the SGA (*n* = 298) and AGA (*n* = 298) preterm groups, with SMD <0.1 to 0.2 for all variables except BW (Table 1).

In the univariate analysis, SGA status among preterm infants was significantly associated with surfactant administration, a history of central line insertion, parenteral nutrition, spontaneous intestinal perforation, and a longer duration of central line indwelling compared with the AGA group (Table 2).

In addition to variables with *p* <0.05, variables with *p* <0.20 in the univariate analysis, including endotracheal intubation at birth, persistent pulmonary hypertension of the newborn, duration of invasive ventilation, and NEC, were included in the multivariate regression model (Table 3). This analysis showed that the composite outcome of mortality or major morbidity, mortality, and mortality or moderate-to-severe BPD were significantly more frequent in SGA preterm infants than in their AGA counterparts.

### 3.2. Very Preterm Infants

Among the 2331 preterm infants, 726 were very preterm, and 95 of these (13.1%) were SGA infants admitted to the NICU. Maternal and neonatal covariates associated with the composite outcome, including GA, male sex, antenatal steroid exposure, and year of birth, together with variables showing SMD >0.1 in the univariate analysis (multifetal gestation, Cesarean delivery, chorioamnionitis, pregnancy-induced hypertension, and 5 min Apgar score), were included in the PSM model. After matching, covariate balance was achieved between the SGA (*n* = 95) and AGA (*n* = 95) very preterm groups, with SMD <0.1 to 0.2 for all variables except BW (Table 4).

In the univariate analysis, SGA status among very preterm infants was significantly associated with surfactant administration, a history of central line insertion, NEC, spontaneous intestinal perforation, and longer durations of central line indwelling and parenteral nutrition compared with the AGA very preterm group (Table 5).

In addition to variables with *p* <0.05, variables with *p* <0.20 in the univariate analysis, including culture-proven sepsis, were included in the multivariate regression model (Table 6). This analysis showed that the composite outcome of mortality or major morbidity, mortality, mortality or moderate-to-severe BPD, and mortality or treated ROP were significantly more frequent in SGA very preterm infants than in their AGA counterparts.

## 4. Discussion

A summary of previous studies comparing outcomes between SGA and AGA infants in preterm and very preterm populations is presented in Table 7. The incidence of the composite outcome among SGA and AGA very preterm infants was 44.3% and 36.1% in Canada [5], 55.0% and 33.4% in the USA [6], and 53.4% and 28.6% in India [3]. In the present study, the corresponding rates were 51.6% and 28.4%, respectively.

Previous studies suggest that outcomes in SGA preterm infants vary according to several key factors: (1) healthcare resources and geographic location, including Canada [5,8], the USA [6,9], Taiwan [11], India [3,10], Ethiopia [4], and Brazil [7]; (2) study period, with data collected before the 2000s [8,9], during the 2000s [5], and throughout the 2010s [3,4,6,7,10,11]; (3) degree of prematurity, ranging from preterm [4,7,10] to very preterm [3,5,6,9,11] and extremely preterm [8] infants; (4) sample size and study setting, including large-scale databases or registries [4,5,6,11] versus single-center cohorts [7,8,9,10]; (5) statistical methodology, such as GA matching [4,9], logistic regression [5,6,11], and PSM [3]; and (6) the definition of composite outcomes, which differs across studies. For example, the composite outcome in the present study is consistent with that used in the USA study [6], whereas other studies included NEC stage II–III [5] or a combination of NEC stage II–III and culture-proven sepsis [3].

The composite outcome rate among SGA very preterm infants in the present study was 51.6%, which is comparable to rates reported in the USA during 2010–2016 (55.0%) [6] and in India during 2015–2019 (53.4%), although the latter study also included both NEC and culture-proven sepsis [3]. In contrast, the incidence of composite outcomes among AGA very preterm infants in this study (28.4% during 2014–2025) was similar to that reported in India (28.6% during 2015–2019) [3], but lower than those reported in Canada (36.1% during 2003–2008) [5] and the USA (33.4% during 2010–2016) [6]. In the contemporary era, the lower incidence of composite outcomes among AGA very preterm infants likely reflects advances in neonatal care. However, these outcomes remain persistently high among SGA very preterm infants. Further research is therefore needed to optimize both antenatal and postnatal care strategies for this population.

The mortality rate among SGA very preterm infants in this study was higher than that among their AGA counterparts, consistent with the reports from Canada [5] and Taiwan [11]. In addition, the incidence of BPD or severe ROP was higher in SGA infants than in AGA infants among extremely preterm infants in Canada [8] and very preterm infants in Canada [5] and Taiwan [11]. We hypothesize that angiogenic dysregulation may represent a shared mechanism underlying BPD and ROP in SGA infants. The same placental insufficiency that restricts somatic growth may also impair microvascular development in the retina and pulmonary capillary bed.

A major strength of this study is the evaluation of composite outcomes in SGA preterm and very preterm infants in a resource-limited setting using both PSM and multivariate analysis. Within the database, PSM was used to identify the most comparable AGA controls for the SGA group. This approach ensured that the SGA and AGA cohorts were balanced with respect to maternal and neonatal baseline characteristics at birth, including GA, sex, antenatal steroid exposure [3], and year of birth to account for potential differences in co-interventions over time. By applying PSM, the average treatment effect on the treated more specifically reflects the impact of SGA status in this population. In addition, neonatal risk factors identified during admission were included in the multivariable analysis to further adjust for confounders influencing discharge outcomes, such as mortality or major morbidity.

This study has some limitations. First, the sample size was relatively small because the data were derived from a single neonatal unit. Second, our data spans a 12-year period, during which temporal advancements in neonatal intensive care protocols may have occurred. Although we meticulously accounted for this by matching SGA and AGA infants within identical birth years using our propensity score algorithm—thereby ensuring contemporary clinical exposures—we cannot completely rule out subtle variations in practice trends over time across the entire study epoch. Furthermore, BPD was defined in our study using traditional oxygen-based criteria. We acknowledge that more contemporary frameworks, such as the Jensen-based definitions, which classify BPD severity according to the specific mode of respiratory support rather than supplemental oxygen use alone, are increasingly adopted in clinical practice. Because our database captures longitudinal data starting from 2014, the traditional definition was maintained to ensure consistency across the study period. Third, the observational design may have introduced inherent bias and residual confounding. To reduce these effects, risk factors present at birth were matched using PSM, and outcomes were further adjusted using multivariate analysis. Although our multivariate models adjusted for several postnatal clinical factors (such as surfactant administration, NEC, parenteral nutrition, and central line use), we acknowledge that, in a strict causal framework, some of these postnatal variables may act as mediators on the causal pathway between SGA status and adverse outcomes. Consequently, our estimates represent the controlled direct effect of SGA rather than its total causal effect, and the potential for overadjustment bias cannot be entirely excluded. Future prospective studies utilizing formal structural equation modeling or directed acyclic graphs are warranted to precisely disentangle the direct and indirect pathways of SGA-mediated neonatal morbidities. Fourth, the exposure was defined strictly as SGA status according to the Fenton growth charts. We lacked antenatal surveillance data, including serial fetal growth trajectories, Doppler velocimetry, and placental pathology records. Consequently, we were unable to differentiate between constitutionally small infants and those suffering from true pathological fetal growth restriction. Additionally, while excluding absolute BW from our propensity score-matching model was methodologically necessary to prevent severe GA imbalance, absolute BW remained substantially lower in the SGA cohort. We cannot entirely rule out residual confounding by BW itself, as a lower BW directly influences neonatal physiological reserves and susceptibility to adverse outcomes. Consequently, the observed associations should be interpreted as the collective impact of fetal growth restriction and its related BW deficit, rather than a purely isolated effect of SGA status alone, which warrants caution against a strictly causal interpretation. Fifth, although the definition of composite outcomes varies across the literature, we believe that SGA outcomes should be interpreted in the context of the specific outcome definition, degree of prematurity, study period, and AGA comparison group. Finally, we focused on outcomes at hospital discharge. In resource-limited settings, the long-term consequences for SGA very preterm infants, including physical growth, neurodevelopmental outcomes, and educational attainment, require further investigation.

## 5. Conclusions

Preterm infants face significantly higher risks of mortality and major morbidity than full-term infants. Moreover, preterm and very preterm infants born SGA are at even greater risk of mortality and certain major morbidities than those born AGA. Multimodal nutritional and hormonal interventions in SGA preterm infants may help to restore metabolic balance, promote appropriate catch-up growth, and reduce the long-term risk of noncommunicable diseases [20].

## Figures and Tables

**Table 1:** Maternal and neonatal characteristics of preterm infants by small-for-gestational-age (SGA) and appropriate-for-gestational-age (AGA) status before and after propensity score matching.

Maternal and Neonatal Characteristics	Overall Cohort (*n* = 2331)				Propensity Score-Matched Cohort (*n* = 596)			
	SGA (*n* = 298)	AGA (*n* = 2033)	Standardized Mean Difference	*p*-Value	SGA (*n* = 298)	AGA (*n* = 298)	Standardized Mean Difference	*p*-Value
Gestational age, weeks *	34 (31–35)	33 (31–35)	0.073	0.05	34 (31–35)	33 (31–35)	−0.051	0.21
Birth weight, g **	1308 ± 449	1903 ± 621	−1.097	<0.001	1308 ± 449	1916 ± 622	−1.211	<0.001
Multifetal gestation, *n* (%)	89 (29.9)	497 (24.4)	0.122	0.04	89 (29.9)	87 (29.2)	−0.029	0.86
Cesarean section, *n* (%)	267 (89.6)	1561 (76.8)	0.348	<0.001	267 (89.6)	267 (89.6)	0.000	1.00
Male sex, *n* (%)	150 (50.3)	1089 (53.6)	−0.065	0.30	150 (50.3)	154 (51.7)	0.013	0.74
Gestational diabetes mellitus, *n* (%)	26 (8.7)	183 (9.0)	−0.009	0.88	26 (8.7)	25 (8.4)	0.012	0.88
Pregnancy-induced hypertension, *n* (%)	68 (22.8)	289 (14.2)	0.223	<0.001	68 (22.8)	54 (18.1)	0.116	0.16
Antenatal magnesium sulfate, *n* (%)	53 (17.8)	290 (14.3)	0.096	0.11	53 (17.8)	46 (15.4)	0.027	0.44
Antenatal steroids, *n* (%)	162 (54.4)	1069 (52.6)	0.036	0.57	162 (54.4)	161 (54.0)	−0.027	0.93
Chorioamnionitis, *n* (%)	2 (0.7)	26 (1.3)	−0.062	0.37	2 (0.7)	4 (1.3)	−0.093	0.41
5 min Apgar score *	9 (8–9)	9 (8–9)	−0.008	0.73	9 (8–9)	9 (8–9)	0.063	0.51

* Data are presented as median (interquartile range). ** Data are presented as mean ± standard deviation.

**Table 2:** Neonatal risk factors in preterm infants by small-for-gestational-age (SGA) and appropriate-for-gestational-age (AGA) status.

Neonatal Risk Factors	SGA (*n* = 298)	AGA (*n* = 298)	*p*-Value
Endotracheal intubation at birth, *n* (%)	104 (34.9)	125 (41.9)	0.08
Surfactant administration, *n* (%)	37 (12.4)	67 (22.5)	0.001
Meconium aspiration syndrome, *n* (%)	5 (1.7)	2 (0.7)	0.25
Pneumothorax, *n* (%)	3 (1.0)	7 (2.3)	0.20
Persistent pulmonary hypertension of the newborn, *n* (%)	5 (1.7)	11 (3.7)	0.13
Ventilator-associated pneumonia, *n* (%)	5 (1.7)	5 (1.7)	1.00
Duration of invasive ventilation, d *	0 (0–2)	0 (0–3)	0.12
Duration of invasive and noninvasive ventilation, d *	2 (0–8)	2 (0–8)	0.41
Central line-associated bloodstream infection, *n* (%)	8 (4.3)	6 (2.8)	0.42
History of central line insertion, *n* (%)	176 (59.1)	147 (49.3)	0.02
Duration of central line indwelling, d *	4 (0–10)	0 (0–9)	0.03
History of parenteral nutrition, *n* (%)	169 (56.7)	124 (41.6)	<0.001
Duration of parenteral nutrition, d *	10 (5–17)	9 (6–15)	0.99
Culture-proven sepsis, *n* (%)	12 (4.0)	9 (3.0)	0.51
Patent ductus arteriosus, *n* (%)	50 (16.8)	52 (17.4)	0.83
Necrotizing enterocolitis (stage II–III), *n* (%)	18 (6.0)	8 (2.7)	0.05
Spontaneous intestinal perforation, *n* (%)	4 (1.3)	1 (0.3)	0.045
Phototherapy, *n* (%)	192 (64.4)	189 (63.4)	0.80

* Data are presented as median (interquartile range).

**Table 3:** Neonatal outcomes in preterm infants with small-for-gestational-age (SGA) versus appropriate-for-gestational-age (AGA): univariate and multivariate analyses.

Outcomes	SGA (*n* = 298)	AGA (*n* = 298)	Univariate Analysis		Multivariate Analysis	
			RR or RD (95% CI) *	*p*-Value	Adjusted RR or RD (95% CI) **	*p*-Value
Composite outcome of mortality or major morbidity	54 (18.1)	31 (10.4)	1.74 (1.15, 2.63)	0.008	1.89 (1.18, 3.02)	0.008
Mortality	28 (9.4)	9 (3.0)	3.11 (1.49, 6.48)	0.002	3.53 (1.57, 7.95)	0.002
Survivors with major morbidity	26/270 (9.6)	22/289 (7.6)	1.27 (0.78, 2.07)	0.34	1.29 (0.75, 2.23)	0.36
Mortality or moderate-to-severe bronchopulmonary dysplasia	53 (17.8)	27 (9.1)	1.96 (1.27, 3.03)	0.002	2.13 (1.30, 3.48)	0.003
Mortality or severe neurological injury ^#^	14/157 (8.9)	15/111 (13.5)	0.66 (0.33, 1.31)	0.24	0.96 (0.42, 2.16)	0.91
Mortality or treated retinopathy of prematurity ^#^	31/105 (29.5)	13/65 (20.0)	1.48 (0.84, 2.61)	0.18	1.54 (0.77, 3.06)	0.22
Length of hospital stay, d	16 (10–31)	14 (8–22)	5.17 (0.90, 9.45)	0.02	1.24 (−1.41, 3.89)	0.36
Daily hospital cost, USD	133 (110–182)	133 (100–188)	1.58 (−13.34, 16.49)	0.84	9.81 (−1.06, 20.68)	0.08

Values are presented as *n* (%) unless otherwise indicated. Length of hospital stay and daily hospital cost are presented as median (interquartile range). CI, confidence interval; RD, risk difference; RR, risk ratio. * Length of hospital stay and daily hospital cost are presented as risk differences; all other outcomes are presented as risk ratios. ** Adjusted for endotracheal intubation at birth, surfactant administration, persistent pulmonary hypertension of the newborn, duration of invasive ventilation, history of central line insertion, duration of central line indwelling, history of parenteral nutrition, necrotizing enterocolitis, and spontaneous intestinal perforation. ^#^ Only infants with relevant indications who underwent evaluation were included.

**Table 4:** Maternal and neonatal characteristics of very preterm infants by small-for-gestational-age (SGA) and appropriate-for-gestational-age (AGA) status before and after propensity score matching.

Maternal and Neonatal Characteristics	Overall Cohort (*n* = 726)				Propensity Score-Matched Cohort (*n* = 190)			
	SGA (*n* = 95)	AGA (*n* = 631)	Standardized Mean Difference	*p*-Value	SGA (*n* = 95)	AGA (*n* = 95)	Standardized Mean Difference	*p*-Value
Gestational age, weeks *	29 (28–30)	29 (28–30)	0.006	0.99	29 (28–30)	29 (27–31)	0.093	0.51
Birth weight, g **	785 ± 184	1220 ± 354	−1.543	<0.001	785 ± 184	1225 ± 396	−1.426	<0.001
Multifetal gestation, *n* (%)	19 (20.0)	180 (28.5)	−0.199	0.08	19 (20.0)	19 (20.0)	0.000	1.00
Cesarean section, *n* (%)	86 (90.5)	455 (72.1)	0.485	<0.001	86 (90.5)	84 (88.4)	0.068	0.64
Male sex, *n* (%)	53 (55.8)	342 (54.2)	0.032	0.77	53 (55.8)	55 (57.9)	−0.042	0.77
Gestational diabetes mellitus, *n* (%)	8 (8.4)	48 (7.6)	0.030	0.78	8 (8.4)	10 (10.5)	−0.072	0.62
Pregnancy-induced hypertension, *n* (%)	36 (37.9)	125 (19.8)	0.406	<0.001	36 (37.9)	35 (36.8)	0.022	0.88
Antenatal magnesium sulfate, *n* (%)	26 (27.4)	145 (23.0)	0.101	0.35	26 (27.4)	33 (34.7)	−0.159	0.27
Antenatal steroids, *n* (%)	70 (73.7)	482 (76.4)	−0.062	0.57	70 (73.7)	67 (70.5)	0.070	0.63
Chorioamnionitis, *n* (%)	0 (0)	13 (2.1)	−0.205	0.16	0 (0)	0 (0)	-	-
5 min Apgar score *	8 (6–9)	8 (6–9)	−0.111	0.08	8 (6–9)	8 (6–9)	−0.038	0.57

* Data are presented as median (interquartile range). ** Data are presented as mean ± standard deviation.

**Table 5:** Neonatal risk factors in very preterm infants by small-for-gestational-age (SGA) and appropriate-for-gestational-age (AGA) status.

Neonatal Risk Factors	SGA (*n* = 95)	AGA (*n* = 95)	*p*-Value
Endotracheal intubation at birth, *n* (%)	72 (75.8)	68 (71.6)	0.51
Surfactant administration, *n* (%)	34 (35.8)	48 (50.5)	0.04
Pneumothorax, *n* (%)	2 (2.1)	4 (4.2)	0.41
Persistent pulmonary hypertension of the newborn, *n* (%)	5 (5.3)	3 (3.2)	0.47
Ventilator-associated pneumonia, *n* (%)	5 (5.3)	3 (3.2)	0.47
Duration of invasive ventilation, d *	3 (1–10)	2 (0–6)	0.31
Duration of invasive and noninvasive ventilation, d *	11 (4–42)	11 (3–37)	0.80
Central line-associated bloodstream infection, *n* (%)	7 (7.7)	5 (5.6)	0.58
History of central line insertion, *n* (%)	92 (96.8)	82 (86.3)	0.009
Duration of central line indwelling, d *	14 (7–31)	10 (5–21)	0.01
History of parenteral nutrition, *n* (%)	79 (83.2)	77 (81.1)	0.71
Duration of parenteral nutrition, d *	16 (10–33)	11 (7–21)	0.002
Culture-proven sepsis, *n* (%)	11 (11.6)	5 (5.3)	0.12
Patent ductus arteriosus, *n* (%)	40 (42.1)	38 (40.0)	0.77
Necrotizing enterocolitis (stage II–III), *n* (%)	18 (18.9)	3 (3.2)	<0.001
Spontaneous intestinal perforation, *n* (%)	4 (4.2)	0 (0)	0.04
Phototherapy, *n* (%)	80 (84.2)	82 (86.3)	0.68

* Data are presented as median (interquartile range).

**Table 6:** Neonatal outcomes in very preterm infants with small-for-gestational-age (SGA) versus appropriate-for-gestational-age (AGA) status: univariate and multivariate analyses.

Outcomes	SGA (*n* = 95)	AGA (*n* = 95)	Univariate Analysis		Multivariate Analysis	
			RR or RD (95% CI) *	*p*-Value	Adjusted RR or RD (95% CI) **	*p*-Value
Composite outcome of mortality or major morbidity	49 (51.6)	27 (28.4)	1.81 (1.25, 2.64)	0.002	1.81 (1.02, 3.23)	0.04
Mortality	25 (26.3)	8 (8.4)	3.13 (1.49, 6.57)	0.003	3.23 (1.09, 9.62)	0.04
Survivors with major morbidity	24/70 (34.3)	19/87 (21.8)	1.57 (0.94, 2.62)	0.09	1.61 (0.76, 3.41)	0.22
Mortality or moderate-to-severe bronchopulmonary dysplasia	48 (50.5)	23 (24.2)	2.09 (1.39, 3.14)	<0.001	2.03 (1.10, 3.72)	0.02
Mortality or severe neurological injury ^#^	13/78 (16.7)	10/72 (13.9)	1.20 (0.56, 2.56)	0.64	1.46 (0.53, 4.02)	0.46
Mortality or treated retinopathy of prematurity ^#^	28/76 (36.8)	10/64 (15.6)	2.36 (1.24, 4.48)	0.01	2.62 (1.03, 6.65)	0.04
Length of hospital stay, d	44 (24–75)	37 (19–58)	8.25 (−2.03, 18.54)	0.12	−0.12 (−7.32, 7.08)	0.98
Daily hospital cost, USD	195 (154–288)	174 (131–203)	38.23 (7.42, 69.04)	0.02	31.38 (−0.72, 63.49)	0.06

Values are presented as *n* (%), except for length of hospital stay and daily hospital cost, which are presented as median (interquartile range). CI, confidence interval; RD, risk difference; RR, risk ratio. * Length of hospital stay and daily hospital cost are presented as risk differences; all other outcomes are presented as risk ratios. ** Adjusted for surfactant administration, history of central line insertion, duration of central line indwelling, duration of parenteral nutrition, culture-proven sepsis, necrotizing enterocolitis, and spontaneous intestinal perforation. ^#^ Only infants with indications who underwent evaluation were included.

**Table 7:** Summary of previous studies comparing preterm and very preterm small-for-gestational-age (SGA) infants with appropriate-for-gestational-age (AGA) infants.

Country [Reference]	Study Period	GA, Weeks	SGA, *n*	AGA, *n*	Method	Outcomes
Canada [8]	1983–1992	<27	37	147	Retrospective	Higher rates of failed medical closure of patent ductus arteriosus, BPD, ROP (stage ≥ III), and prolonged oxygen supplementation and ventilatory support.
USA [9]	1985–1988	<32	27	27	Prospective, GA-matched	No difference in neonatal course. At 3 years of age, SGA infants had lower weight, shorter height, and poorer development.
Canada [5]	2003–2008	<33	1249	10,660	Retrospective registry study, logistic regression	Higher odds of mortality, NEC, BPD, and severe ROP. Lower odds of survival without major morbidity (55.7% vs. 63.9%; aOR, 0.50; 95% CI, 0.43–0.58) and respiratory distress syndrome. Longer neonatal intensive care unit stay and longer duration of ventilation.
USA [6]	2010–2016	<32	743	5965	Retrospective database study, logistic regression	Increased risk of the composite outcome of mortality or major morbidity (55.0% vs. 33.4%) and death.
Taiwan [11]	2012–2017	<33	1005	3238	Prospective database study, subgroup analysis, logistic regression	Increased risk of neonatal mortality, BPD (at GA 27–29 and 30–32 weeks), and severe ROP (at GA 24–26 and 27–29 weeks). Increased risk of neurodevelopmental impairment and growth status below the 10th percentile at 2 years.
India [3]	2015–2019	<32	148	444	Retrospective, propensity score-matched	Increased odds of the composite outcome of mortality or major morbidity (53.4% vs. 28.6%; aOR, 2.99; 95% CI, 1.96–4.57), NEC stage II–III, hypoglycemia, and anemia.
India [10]	2015–2018	<35	59	119	Cross-sectional	Increased risk of NEC stage II–III, underweight, stunting, and delayed motor and mental development.
Ethiopia [4]	2016–2018	28–36	668	668	Multisite, GA-matched	Increased risk of hypoglycemia, NEC, polycythemia, late-onset neonatal sepsis, and prolonged hospitalization.
Brazil [7]	2017	<37	27	102	Retrospective cohort	Associated with intraventricular hemorrhage, ROP, and patent ductus arteriosus.
This Study	2014–2025	<37	298	298	Propensity score-matched, multivariate analysis	Increased risk of the composite outcome of mortality or major morbidity (18.1% vs. 10.4% for GA <37 weeks; 51.6% vs. 28.4% for GA <32 weeks), mortality, mortality or moderate-to-severe BPD, and mortality or treated ROP.
		<32	95	95		

aOR, adjusted odds ratio; BPD, bronchopulmonary dysplasia; CI, confidence interval; GA, gestational age; NEC, necrotizing enterocolitis; ROP, retinopathy of prematurity; vs., versus.

## Data Availability

The raw data supporting the conclusions of this study are available from the authors upon request. These data are not publicly available because of privacy concerns.

## Abbreviations

The following abbreviations are used in this manuscript:

AGA	Appropriate-for-gestational-age
BPD	Bronchopulmonary dysplasia
GLM	Generalized linear model
LGA	Large-for-gestational-age
NEC	Necrotizing enterocolitis
PSM	Propensity score matching
ROP	Retinopathy of prematurity
SGA	Small-for-gestational-age
SMD	Standardized mean difference
